# *cis*-DA-dependent dispersion by *Pseudomonas aeruginosa* biofilm and identification of *cis*-DA-sensory protein DspS

**DOI:** 10.1128/mbio.02570-23

**Published:** 2023-11-28

**Authors:** Manmohit Kalia, Diana Amari, David G. Davies, Karin Sauer

**Affiliations:** 1 Department of Biological Sciences, Binghamton University, Binghamton, New York, USA; 2 Binghamton Biofilm Research Center, Binghamton University, Binghamton, New York, USA; University of Washington, Seattle, Washington, USA

**Keywords:** signal perception, dispersion, *cis*-2-decenoic acid, DSF, RpfC, DspS, mechanism, dispersion pathway, c-di-GMP

## Abstract

Native dispersion, the terminal stage in biofilm development, is characterized by the active escape of cells from a biofilm, leaving behind central voids or hollow structures. However, much of what is known about the dispersion mechanism stems from results obtained in experiments using exogenously added dispersion cues such as nitric oxide (NO) and glutamate. To begin exploring the mechanism of native (endogenous) dispersion by *Pseudomonas aeruginosa* PAO1 biofilms, we examined the similarities between dispersion exogenously induced with NO and the previously reported native dispersion inducer, *cis*-2-decenoic acid (*cis*-DA), as well as native dispersion. Induction of dispersion with *cis*-DA was similar to induction with NO, with a significant reduction in cyclic dimeric guanosine monophosphate levels compared with uninduced cells but increased expression of *pelA*, *pslG*, *endA*, and *eddA*. Of those factors known to contribute to *P. aeruginosa* biofilm dispersion induced by glutamate and NO, only BdlA*,* AmrZ, RbdA, and DipA were shown to contribute to dispersion induced with *cis*-DA. The above factors were also shown to contribute to dispersion when no exogenous inducer was added, as indicated by microcolony void formation, a hallmark of native (endogenous) biofilm dispersion. Interestingly, phosphodiesterase PA2133, the previously reported dispersion sensors (NbdA, MucR, and NicD), and a predicted *cis*-DA sensor PA4892 played no detectable role in native or *cis*-DA-dependent dispersion. Instead, we show that *cis*-DA signal sensing by *P. aeruginosa* required the sensor/response regulator hybrid DspS (PA4112), with inactivation of *dspS* impairing *cis*-DA-induced and native dispersion in two *P. aeruginosa* strains, PAO1 and PA14. Overall, our findings indicate that while sensing of *cis*-DA and dispersion cues such as NO and glutamate are distinct, the downstream mechanisms leading to the liberation of biofilm cells and, thus, dispersion rely on a shared pathway.

**IMPORTANCE:**

Dispersion is an essential stage of the biofilm life cycle resulting in the release of bacteria from a biofilm into the surrounding environment. Dispersion contributes to bacterial survival by relieving overcrowding within a biofilm and allowing dissemination of cells into new habitats for colonization. Thus, dispersion can contribute to biofilm survival as well as disease progression and transmission. Cells dispersed from a biofilm rapidly lose their recalcitrant antimicrobial-tolerant biofilm phenotype and transition to a state that is susceptible to antibiotics. However, much of what is known about this biofilm developmental stage has been inferred from exogenously induced dispersion. Our findings provide the first evidence that native dispersion is coincident with reduced cyclic dimeric guanosine monophosphate levels, while also relying on at least some of the same factors that are central to the environmentally induced dispersion response, namely, BdlA, DipA, RbdA, and AmrZ. Additionally, we demonstrate for the first time that cis-DA signaling to induce dispersion is attributed to the two-component sensor/response regulator DspS, a homolog of the DSF sensor RpfC. Our findings also provide a path toward manipulating the native dispersion response as a novel and highly promising therapeutic intervention.

## INTRODUCTION

Biofilms are structured communities of bacterial cells enmeshed within a self-produced polymeric matrix that is adherent to inert or living surfaces or can be present in the form of aggregates of microorganisms, such as within a tissue or in suspension in a fluid (1–3). The ability to form a biofilm is a common trait of a diverse array of microbes, including lower-order eukaryotes, with biofilms being the predominant mode of bacterial growth in nature (4). The sessile lifestyle affords bacteria multiple protective advantages, allowing bacteria to remain within a favorable environmental niche or host. Compared to free-swimming bacteria, biofilms are better adapted to withstand nutrient deprivation, pH changes, oxygen radicals, biocides, and antimicrobial agents (5). However, as a biofilm grows, the community becomes stratified, resulting in the interior of biofilms experiencing reduced nutrient availability, increased exposure to waste products and toxins, and gradients impacting oxygen, pH, and other environmental parameters. To escape these threatening conditions, biofilm cells have evolved mechanisms to escape the sessile mode of growth by liberating themselves from the matrix and reverting back to a free-living lifestyle in a physiologically regulated process known as a biofilm dispersion response (6–10).

Activation of a dispersion response requires signals or cues that lead to a cascade of events resulting in the release of cells from the biofilm. Depending on whether the trigger originates from the microbes (endogenous) or is an environmental cue (exogenous), two types of dispersion can be distinguished: native (endogenous) and environmentally induced (exogenous) dispersion. Both types of dispersion are characterized by single cells actively escaping from the biofilm, with the resulting dispersed cell populations sharing similar phenotypes, including increased susceptibility to antimicrobial agents (11–14). However, native dispersion generally results in the evacuation of cells from the interior of microcolonies, leaving central voids (6, 11, 15, 16), while induced dispersion leaves behind biofilms that are eroded from the outside (8, 11, 17).

Environmentally induced dispersion occurs in response to factors that are present in the surrounding environments, including nutrients (glucose, glutamate, succinate, and citrate), peptide signals, nitric oxide (NO), heavy metals, and starvation and oxygen-limiting conditions (15, 18–22). In *Pseudomonas aeruginosa*, environmental cues are sensed by membrane-bound receptors such as NicD and NbdA (23, 24). Signal sensing results in post-translational modification and subsequent activation of the chemosensory protein BdlA, a process resulting in the non-processive proteolysis of BdlA and requiring increased cyclic dimeric guanosine monophosphate (c-di-GMP) levels (via the diguanylate cyclase GcbA), protease ClpP, and the chaperone ClpD (17, 24, 25). Active BdlA, in turn, recruits and activates the phosphodiesterases DipA and RbdA to ultimately reduce cellular c-di-GMP levels. The biofilm matrix is composed of various extracellular polymers and may include polysaccharides, eDNA, and proteins (26, 27). To liberate themselves from the enmeshing matrix, dispersing biofilm cells make use of degradative enzymes such as hydrolases and endonucleases. An additional player includes the transcriptional regulator AmrZ, which directly or indirectly regulates several genes encoding components known to be associated with the dispersion process (28, 29). Specifically, AmrZ represses the diguanylate cyclase-encoding gene *gcbA* (*PA4843*) (30), *fleQ* transcription and motility (29–31), and production of the extracellular polysaccharide Psl (28, 30) while activating alginate production (32) and twitching motility (30, 33). RNA-seq and ChIP-seq data indicated that AmrZ positively affected the expression of genes encoding matrix-degrading enzymes, including *pslG*, *pelA*, *endA*, *eddA*, and *cdrA* (30).

In contrast, native dispersion has been reported to occur in response to self-synthesized signaling molecules or cues whose concentration is impacted by the steep gradients within the biofilm (10). When biofilm microcolonies are small, these molecules are typically transported away from the biofilm bacteria and are diluted in the extracellular milieu. However, when the overall volume of a microcolony increases, the ability to wash out these cues diminishes until a threshold is reached and the cue induces a dispersion response. In *P. aeruginosa*, the native dispersion inducer has been identified as the fatty acid signaling molecule *cis*-2-decenoic acid (*cis*-DA) (11). Production of *cis*-DA by *P. aeruginosa* requires an enoyl-CoA synthetase encoded by *dspI* (PA14_54640, a PA0745 ortholog), with *dspI* inactivation resulting in significantly reduced dispersion events and defective swarming motility (34). Transcriptomic profiling indicated that *cis*-DA affects the expression of 666 genes (35). Purified *cis*-DA isolated from cultures of *P*. *aeruginosa* has been shown to induce biofilm dispersion in a range of Gram-negative and Gram-positive bacteria and yeast (11).

Like *cis*-DA, the diffusible factor DSF is a fatty acid signaling molecule. DSF has been previously reported to coordinate virulence factor production by the bacterial pathogen *Xanthomonas campestris* (36, 37). DSF signal sensing by *X. campestris* requires the *rpf* gene cluster (36, 37). Production of DSF requires the enoyl–CoA hydratase RpfF (37–39). Sensing of the DSF signal and signal transduction has been attributed to a two-component sensory transduction system comprising the hybrid sensor kinase RpfC and the response regulator RpfG (37–39). DSF sensing is believed to result in RpfC autophosphorylation and subsequent phosphotransfer to RpfG. RpfG lacks a DNA-binding domain but instead harbors an HD-GYP domain that exhibits phosphodiesterase activity capable of degrading c-di-GMP to GMP (37, 39–41). Phosphorylation is thought to activate RpfG for c-di-GMP degradation. RpfC/RpfG link sensing of the cell–cell signal DSF to alteration in the cellular level of c-di-GMP. However, it is interesting to note that the *rpf* gene cluster is absent in *P. aeruginosa*, with DspI only sharing 30% homology with RpfF. Using microarray analysis, Rahmani-Badi et al. (35) proposed PA4982-PA4983 encoding via a two-component system to be involved in *cis*-DA signal sensing. However, there is no experimental evidence for *cis*-DA being sensed via PA4982-PA4983.

The above findings underscore how little is known about the mechanism of native dispersion and/or dispersion in response to *cis*-DA. This raises the question of how *P. aeruginosa* perceives *cis*-DA, how *cis*-DA signal sensing is relayed, and how signaling of *cis*-DA induces dispersion in *P. aeruginosa* biofilms. This study aimed to determine whether endogenous dispersion and dispersion induced by *cis*-DA, a native dispersion inducer, share any or all of the same mechanistic components as those known to be involved in dispersion induced by NO or glutamate. Furthermore, we wished to identify the signal sensor for recognition and signal transduction, inducible by interaction with *cis*-DA.

## RESULTS

### Biofilms by *P. aeruginosa* PAO1 disperse in response to the native dispersion cue *cis*-2-decenoic acid

**Fig 1:**
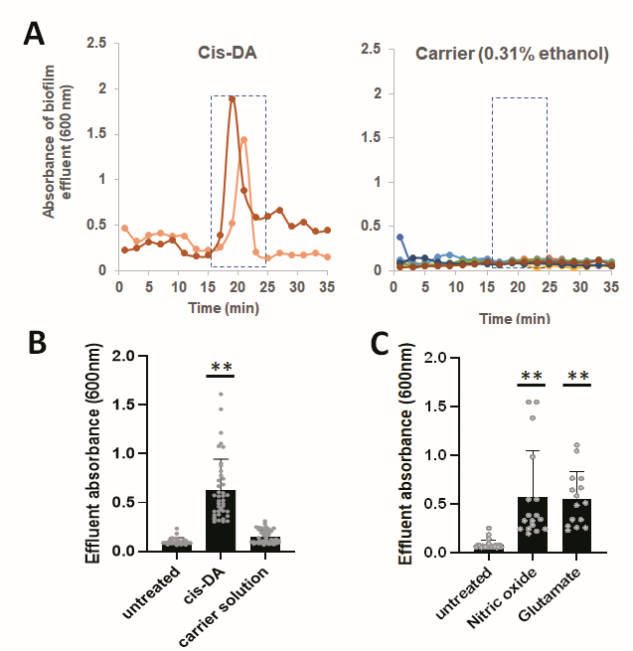
Dispersion response of biofilms by *P. aeruginosa* PAO1 following exposure to *cis*-DA (**A**) Dispersion response of 5-day-old *P. aeruginosa* PAO1 biofilms grown in tube reactors under flowing conditions following exposure to 310 nM *cis*-DA or carrier solution (0.31% ethanol) alone. Post-induction of dispersion, effluents from tube reactors were collected for 35 min in 1-min intervals and the absorbance was determined by spectrophotometry at 600 nm. Individual data points represent absorbance of effluents collected in 1-min intervals. Spikes in the absorbance of the effluent are indicative of positive dispersion responses. Dispersion assays were performed in triplicate using four technical replicates each, but only representative dispersion responses from biological replicates are shown by colored lines. (**B**) Quantitative analysis of the dispersion response following exposure to 310 nM *cis*-DA or carrier solution. Individual data points represent absorbance values of biofilm effluents collected 15 to 25 min post-exposure to *cis*-DA or carrier solution (see dashed boxes shown in panel **A**). Absorbance readings of effluents of untreated biofilms collected between 15 and 25 min were used as control (untreated). (**C**) Quantitative analysis of the dispersion response post-exposure to nitric oxide and 18 mM glutamate. Sodium nitroprusside (500 µM) was used as a source of nitric oxide. Individual data points represent absorbance values of effluents collected 15 to 25 min post-exposure of biofilms to glutamate or nitric oxide or of untreated biofilms. **, significantly different from untreated biofilms (*P* < 0.01), as determined by analysis of variance (ANOVA) followed by a Dunnett’s post-hoc test.

The native dispersion inducer by *P. aeruginosa* PA14 has been identified as the fatty acid signaling molecule *cis*-2-decenoic acid (*cis*-DA) (11). The native concentration of *cis*-DA produced by *P. aeruginosa* PA14 grown as laboratory biofilm in continuous culture was found to be 2.5 nM. Moreover, previous findings indicated *cis*-DA was able to induce dispersion at concentrations ranging from 1 nM to 10 mM when exogenously added to the growth medium of 5–6-day-old biofilms formed by *P. aeruginosa* PA14 grown in continuous culture (11); however, concentrations of 310 nM *cis*-DA have routinely been used in dispersion assays (34, 35) and to coincide with the release of 33%–55% of the biofilm population, as determined by CFU (11, 34). Given that *cis*-DA has been reported to induce dispersion by *P. aeruginosa* PA14 biofilms (11, 34, 35), we first asked whether *cis*-DA, when exogenously added, can likewise disperse biofilms formed by *P. aeruginosa* PAO1. We used biofilms grown for 5 days in tube reactors under continuous flow conditions and subsequently induced dispersion by the sudden addition of 310 nM *cis*-DA to the growth medium. We collected effluents from the biofilm tube reactor post-induction of dispersion, with dispersion apparent by a sharp increase in the absorbance at 600 nm of the effluent as early as 15–20 min following induction, compared with untreated biofilms (15, 17, 23–25, 42). Under the conditions tested, biofilms of *P. aeruginosa* PAO1 demonstrated a sharp increase in absorbance at 600 nm in the effluent within 15–20 min after switching the growth medium to medium containing *cis*-DA (Fig. 1A), a response which was absent in biofilms exposed to the carrier solution alone or biofilms left untreated (Fig. 1A). To quantitate the dispersion response, we evaluated the absorbance of effluents of untreated biofilms and biofilms exposed to *cis*-DA or carrier solution, collected between 15 and 25 min (Fig. 1A). The quantitative analysis of the dispersion response indicated exposure to exogenously added *cis*-DA to coincide with a significant increase in the effluent absorbance (Fig. 1B) and, thus, the number of cells released from the biofilms. Moreover, the response (timing and average release of cells, change in absorbance relative to control biofilms) noted post-exposure to *cis*-DA was comparable to the dispersion response noted to glutamate and nitric oxide (Fig. 1C; Fig. S1). Previous findings indicated dispersion in response to glutamate to coincide with the release of up to 50% of the biofilm population, as determined by CFU (15), and up to 70% of the *P. aeruginosa* PAO1 and PA14 biofilm population, as determined by confocal microscopy and COMSTAT analysis (23, 24, 43). Likewise, COMSTAT analysis indicated dispersion in response to nitric oxide to reduce the biomass of biofilms formed by *P. aeruginosa* PAO1 and PA14 by 50%–80% (23, 24, 43). Overall, our findings are in agreement with Davies and Marques (11) and strongly suggest biofilms formed by *P. aeruginosa* PAO1 disperse in response to exogenously added *cis*-DA. Additionally, our findings suggested dispersion in response to *cis*-DA to be similar to dispersion in response to exogenous glutamate and nitric oxide.

### Dispersion in response to the native dispersion inducer *cis*-DA coincides with reduced c-di-GMP levels of the dispersing population

Dispersion in response to nutrients and nitric oxide has previously been reported to coincide with a decrease in cellular levels of the intracellular signaling molecule bis-(3′−5′)-cyclic dimeric guanosine monophosphate (c-di-GMP) (17, 23–25, 42–47). Moreover, both phosphodiesterases DipA and RbdA are responsible for the modulation of c-di-GMP upon induction of dispersion in response to nutrient cues and nitric oxide (43, 48). Given the similarities in the dispersion response following exposure of *P. aeruginosa* biofilms to *cis*-DA, glutamate, and nitric oxide, we next asked if dispersion in response to the native dispersion inducer *cis*-DA likewise contributes to the modulation of the cellular levels of c-di-GMP in the dispersed cell population. To quantitative c-di-GMP levels, we made use of an unstable GFP reporter [P*_cdrA_::gfp*(ASV)] for which the fluorescence intensity is directly proportional to the concentration of intracellular c-di-GMP (49).

**Fig 2:**
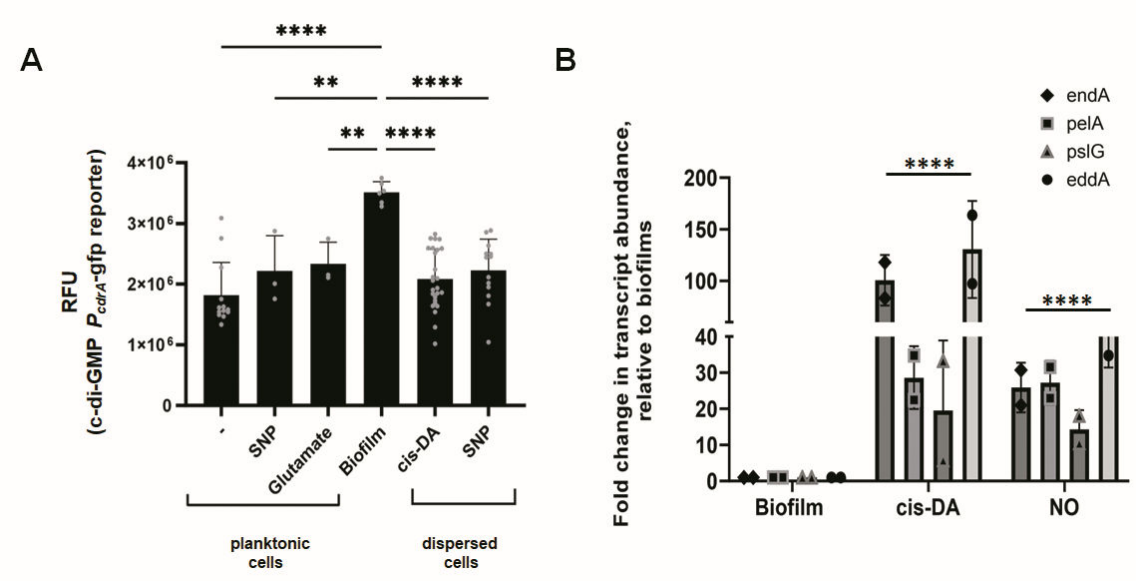
Changes in c-di-GMP level and transcript abundance of genes encoding matrix-degrading enzymes in response to *cis*-DA-induced dispersion. (**A**) Relative levels of intracellular c-di-GMP. Planktonic cells were grown in Vogel and Bonner citrate minimal medium (VBMM) to the exponential phase while biofilms were grown for 5 days in fivefold diluted VBMM in tube reactors under flowing conditions. Dispersed cells were obtained by collecting biofilm effluents post-exposure of biofilms to nitric oxide or *cis*-DA. *P. aeruginosa* PAO1 harboring the unstable c-di-GMP reporter pCdrA::*gfp*(ASV) was used for all experiments. The relative fluorescence unit (RFU) was normalized to A600nm. Experiments were performed using biological triplicates. Individual data points represent normalized RFU values of replicates. Error bars indicate standard deviations. ** and ****, significantly different from biofilms, *P* < 0.05 and *P* < 0.001, respectively, as determined by ANOVA followed by a Dunnett’s post-hoc test. (**B**) Transcript abundance of genes encoding matrix-degrading enzymes. RNA was obtained from biofilms grown for 5 days in fivefold diluted VBMM in tube reactors under flowing conditions. RNA was obtained from dispersed cells by collecting biofilm effluents post-exposure of biofilms to nitric oxide or cis-DA. The transcript abundance of genes encoding endonucleases EndA and EddA and hydrolases PelA and PslG was determined by quantitative reverse transcriptase PCR (qRT-PCR). Experiments were performed using biological duplicates with two technical replicates. Individual data points represent average fold changes in transcript abundance. Error bars indicate standard deviations. ****, significantly different from respective genes under biofilm growth conditions (*P* < 0.001), as determined by ANOVA followed by a Dunnett’s post-hoc test.

We first determined c-di-GMP levels in planktonic and biofilm cells and cells dispersed from the biofilm in response to *cis*-DA. Dispersed cells obtained in response to nitric oxide were used as controls. As anticipated, significant differences in c-di-GMP levels were noted between *P. aeruginosa* PAO1 planktonic and biofilm cells (Fig. 2A). Dispersed cells obtained in response to *cis*-DA were found to harbor cellular levels of c-di-GMP that were reduced relative to (untreated) wild-type biofilms but similar to planktonic cells (Fig. 2A). C-di-GMP levels of dispersed cells obtained in response to nitric oxide were comparable to those found for *cis*-DA dispersed cells (Fig. 2A). It is of interest to note that the absence or presence of *cis*-DA or nitric oxide under planktonic growth conditions had no effect on the levels of c-di-GMP of planktonic cells (Fig. 2A). The findings suggested that similar to nitric oxide-induced dispersion, dispersion in response to the native dispersion cue *cis*-DA coincided with an overall reduction in the cellular c-di-GMP levels.

### Dispersion in response to the native dispersion inducer *cis*-DA coincides with increased expression of genes encoding matrix-degrading enzymes

Biofilms are enmeshed in a biofilm matrix composed of exopolysaccharides, proteins, lipids, and extracellular DNA (eDNA), collectively called the extracellular polymeric substance, representing up to 85% of the total biofilm biomass (27, 50). In turn, dispersion has been linked to matrix degradation to enable the liberation of cells from the enmeshed biofilm structure. This is supported by dispersed cells demonstrating increased degradation of Psl, protein, lipids, and DNA relative to biofilms (45), with biofilms disassembling following exposure to purified glycoside hydrolases including PelA and PslG, and amylases (13, 51, 52) or upon overexpression of genes encoding matrix-degrading enzymes including *endA*, *eddA*, and *eddB* encoding the DNA-degrading enzymes EndA, EddA, and EddB, respectively, as well as *pelA* and *pslG* (53, 54). Additionally, dispersed cells demonstrate increased expression of *pel*, *psl*, *endA*, and *eddAB* (53–55). Interestingly, increased *pel* expression has recently been shown to coincide with the production of cell-free, soluble Pel polysaccharide, with the shift from cell-associated to soluble Pel being due to the glycoside hydrolase activity of PelA (56). The increased hydrolytic activity of PelA is in agreement with the increased abundance of extracellular PelA and PslG upon induction of dispersion (54).

To further characterize dispersion in response to *cis*-DA, we asked whether induction of dispersion by *cis*-DA likewise affects the expression of *pel* and *psl* and genes encoding DNA-degrading enzymes. Genes of interest included *pelA*, *pslG*, *eddA*, and *endA* (53, 54, 57). Biofilms formed by PAO1 and dispersed cells obtained in response to nitric oxide were used as controls. Relative to intact biofilm cells, the transcript abundance of *endA*, *eddA*, *pelA*, and *pslG* was significantly increased in nitric oxide-dispersed cells relative to intact biofilm cells (Fig. 2B). Overall, expression of genes encoding matrix-degrading enzymes was up to 20-fold increased in dispersed cells relative to intact biofilm cells (Fig. 2B). The results are in agreement with previous reports (53, 54). Similar results were obtained for *cis*-DA-dispersed cells (Fig. 2B). However, while the expression of *pelA* and *pslG* was significantly increased relative to biofilm cells, the transcript abundance of *endA* and *eddA* was increased up to 100-fold (Fig. 2B).

Our findings suggest that dispersed cells obtained in response to *cis*-DA induce the expression of *pel*, *psl*, and genes encoding matrix-degrading enzymes in a manner similar to glutamate- or nitric oxide-induced dispersed cells. Moreover, our findings imply that similar to nutrient- or nitric oxide-induced dispersion, dispersion in response to *cis*-DA likewise coincides with matrix degradation.

### Exposure to two dispersion cues, nitric oxide, and *cis*-DA does not have an additive effect on the dispersion response

Dispersion in response to nitric oxide and glutamate has been reported to require the presence of the regulatory proteins AmrZ and BdlA and the two phosphodiesterases, DipA and RbdA. Given the similarity between *cis*-DA-induced and nitric oxide-induced dispersed cells (Fig. 1 and 2), we next asked if the relay of the dispersion signal *cis*-DA and dispersion cues such as nitric oxide proceeds via the same pathway. We reasoned that if both cues are perceived via the same regulatory pathway, exposure to the dispersion signal *cis*-DA would not result in an enhanced dispersion response of biofilms when simultaneously challenged with nitric oxide. However, if the dispersion cues target distinct pathways leading to dispersion, we anticipated an enhanced dispersion response.

**Fig 3:**
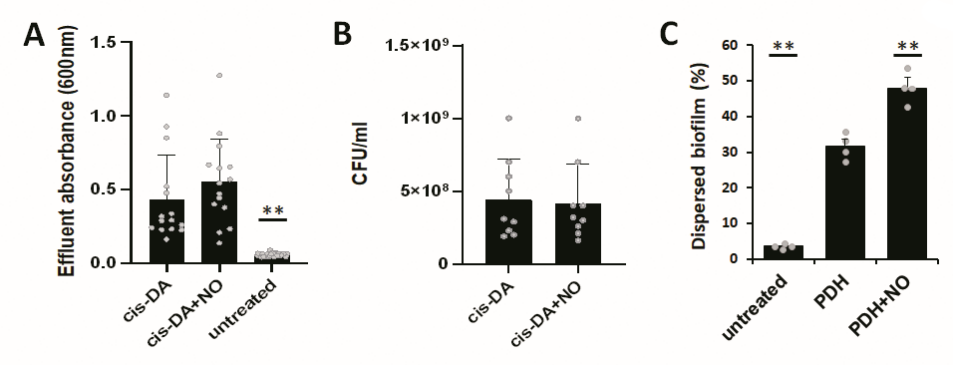
Effect of combining dispersion inducers on the dispersion response. Biofilms by *P. aeruginosa* were grown for 5 days in fivefold diluted VBMM in tube reactors under flowing conditions. (**A and B**) Dispersion was induced after 5 days of growth by the addition of 310 nM *cis*-DA in the absence or presence of nitric oxide to the growth medium (see cis-DA vs cis-DA + NO). Untreated biofilms were used as control. The effluent from tube reactors was collected for 30 min in 1-min intervals, and the absorbance was determined by spectrophotometry at 600 nm. (**A**) Quantitative analysis of the dispersion response post-exposure to 310 nM *cis*-DA in the absence or presence of nitric oxide to the growth medium. Individual data represent absorbance values of effluents collected 15 to 25 min post-exposure to *cis*-DA in the absence or presence of nitric oxide, obtained in triplicate. Error bars represent standard deviations. **, significantly different from untreated biofilms exposed to *cis*-DA alone (*P* < 0.01), as determined by ANOVA followed by a Dunnett’s post-hoc test. (**B**) Number of viable cells, expressed as CFU/mL, in the biofilm effluents post-addition of *cis*-DA in the absence or presence of nitric oxide. Experiments were carried out in triplicates. Individual data represent CFU values of effluents collected 15 to 25 min post-exposure to *cis*-DA in the absence or presence of nitric oxide. Error bars represent standard deviations. (**C**) Biofilms by *P. aeruginosa* were grown for 5 days in fivefold diluted VBMM 24-well plates under semi-batch conditions. Dispersion was induced by the addition of pyruvate dehydrogenase (PDH, plus cofactors) to induce pyruvate-depleting conditions in the absence or presence of nitric oxide. Biofilms only exposed to cofactors were used as control. Dispersed cells were determined by evaluating the number of viable cells in biofilm supernatants. Individual data represent CFU values obtained from triplicate experiments. **, significantly different from biofilms exposed to pyruvate dehydrogenase alone (*P* < 0.01), as determined by ANOVA followed by a Dunnett’s post-hoc test.

To test this hypothesis, we first exposed biofilms to either *cis*-DA alone or a combination of *cis*-DA and nitric oxide. While both treatments resulted in dispersion events, no difference in the dispersion pattern was noted (Fig. S2). Quantitative analysis confirmed exposure of biofilms to *cis*-DA alone or a combination of *cis*-DA and nitric oxide did not result in a significantly different dispersion response (Fig. 3A). Moreover, no significant difference in the number of dispersed cells in response to *cis*-DA alone or a combination of *cis*-DA and nitric oxide was noted (Fig. 3B). The finding indicated co-exposure *cis*-DA and nitric oxide not to have an additive effect on the dispersion response. Moreover, the finding suggested that the dispersion signal *cis*-DA and nitric oxide likely induce dispersion via the same regulatory pathway.

Previous findings indicated depletion of pyruvate from the growth medium to result in biofilm dispersion (58). The dispersion response was found to be dependent on pyruvate fermentation pathway components but independent of proteins previously described to contribute to *P. aeruginosa* biofilm dispersion (58), suggesting pyruvate depletion-induced dispersion to be distinct from, e.g., nitric oxide-induced dispersion. To further support the notion of *cis*-DA and nitric oxide inducing dispersion via the same regulatory pathway, we asked exposure of *P. aeruginosa* biofilms to pyruvate-depleting conditions in the presence of nitric oxide would result in an enhanced dispersion response.

We exposed *P. aeruginosa* biofilms to either pyruvate-depleting conditions alone or in combination with nitric oxide. Relative to untreated biofilms, the addition of pyruvate dehydrogenase (PDH) to induce pyruvate-depleting conditions coincided with a significant increase in the number of cells released from the biofilms (Fig. 3C). Based on viability counts, the respective treatment resulted, on average, in 30% of the biofilm biomass to disperse (Fig. 3C). However, the percentage of biofilms that dispersed increased to 50% when biofilms were exposed to both pyruvate dehydrogenase and nitric oxide (Fig.3C)

**Fig 4:**
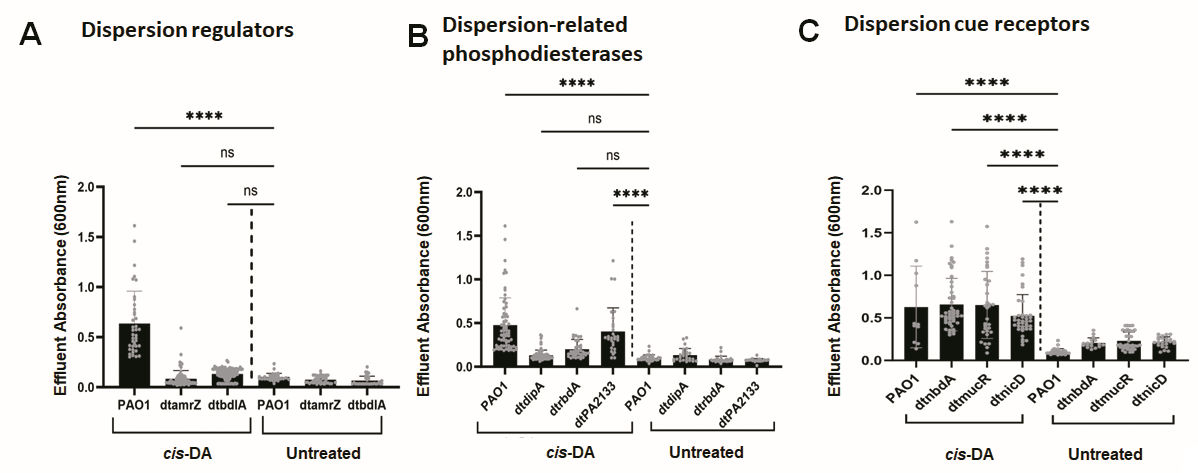
Contribution of factors previously reported to contribute to NO- or glutamate-induced dispersion response to dispersion in response to the exogenously added native dispersion signal *cis*-DA. Biofilms were grown for 5 days in fivefold diluted VBMM in tube reactors under flowing conditions. Dispersion was induced after 5 days of growth by the addition of 310 nM *cis*-DA to the growth medium. Effluent from tube reactors was collected for 30 min in 1-min intervals, and the absorbance was determined by spectrophotometry at 600 nm. Untreated biofilms were used as controls. Experiments were carried out in triplicate. Quantitative dispersion response of biofilms formed by (**A**) dispersion regulator mutant strains dt*bdlA* and dt*amrZ* and (**B**) phosphodiesterase mutant strains dtPA2133, dt*dipA*, and dt*rbdA* and (**C**) dispersion cue receptor mutant strains dt*nicD*, dt*mucR*, and dt*nbdA*. Individual data represent absorbance values of effluents collected 15–25 min post-addition of *cis*-DA obtained in triplicate. Error bars represent standard deviations. * and ****, significantly different from untreated PAO1 biofilms (*P* < 0.05 and *P* < 0.001, respectively), as determined by ANOVA followed by a Dunnett’s post-hoc test. ns, not significant.

### Biofilms by *P. aeruginosa* mutant strains, impaired in their dispersion response to glutamate and nitric oxide, likewise fail to disperse in response to the native dispersion cue *cis*-DA

Dispersion induced in response to glutamate and nitric oxide has been reported to require the phosphodiesterases DipA, RbdA, and PA2133 and the regulatory proteins BdlA (chemotaxis transducer) and AmrZ (17, 25, 42, 43, 48, 59, 60). Given that *cis*-DA and nitric oxide likely induce dispersion via the same regulatory pathway, we next asked if these factors also contribute to dispersion in response to *cis*-DA. We hypothesized that if these factors play a role in *cis*-DA-induced dispersion, biofilms by the respective mutant strains would be impaired in dispersion.

Biofilms by wild-type *P. aeruginosa* and the respective mutant strains were grown for 5 days in tube reactors under continuous flow conditions, after which dispersion was induced by adding *cis*-DA to the growth medium. Post-induction of dispersing-inducing conditions, effluents from the biofilm tube reactor were collected, and the absorbance was determined at 600 nm. No sharp increases in the absorbance (600 nm) in the effluent of biofilms formed by mutants inactivated in genes encoding the regulatory proteins BdlA and AmrZ in response to *cis*-DA were noted (Fig. 4A; Fig. S3A). Likewise, biofilms formed by mutants inactivated in the phosphodiesterase genes *dipA* and *rbdA* failed to disperse (Fig. 4B; Fig. S3B). In contrast, biofilms formed by dtPA2133 dispersed (Fig. 4B; Fig. 3B) in a manner similar to wild-type biofilms (Fig. 1A, B and 4A).

Our findings suggest that similar to glutamate- or nitric oxide-induced dispersion,dispersion in response to the native dispersion inducer *cis*-DA requires the regulatory proteins AmrZ and BdlA as well as the two phosphodiesterases DipA and RbdA (Table 1). However, the native dispersion inducer appears to bypass the need for the phosphodiesterase PA2133 (Table 1).

**TABLE 1:** Overview of dispersion response by *P. aeruginosa* wild-type and mutant strains^*a*^

Strains	Description	Response to nutrients (e.g., succinate, glutamate)	Response to nitric oxide	Response to cis-DA	Void formation^*a*^	Native dispersion (autoinduction)
PAO1		Dispersion (42)	Dispersion (44)	Dispersion (11)	Yes, 80%–92%	Yes
*dtbldA*	Chemotaxis transducer	Impaired (42)	Impaired (44)	Impaired	Reduced, 14%–18%	No
*dtamrZ*	Transcriptional regulator	Impaired (59)	Impaired (59)	Impaired	Reduced, 16%–18%	No
*dtdipA*	Phosphodiesterase	Impaired (43)	Impaired (43)	Impaired	Reduced, 11%–12%	No
*dtrbdA*	Phosphodiesterase	Impaired (48)^a^	Impaired (48),	Impaired	Reduced, 7%–10%	No
dtPA2133	Phosphodiesterase	Dispersion (12), dispersion upon multicopy expression (60)	Dispersion (12), dispersion upon multicopy expression (60)	Dispersion	WT^*c*^-like, 70%–90%	Yes
dt*nicD*	Membrane-bound diguanylate cyclase	Impaired (24)	Dispersion (24, 43)	Dispersion	ND^*b*^	Yes
dt*nbdA*	Membrane-bound phosphodiesterase	Dispersion (23)	Impaired (23)	Dispersion	ND	Yes
dt*mucR*	Membrane bound protein (dual activity, phosphodiesterase, and diguanylate cyclase	Impaired (23)	Impaired (23)	Dispersion	ND	Yes
dt*dspS*	Membrane-bound sensor/response regulator hybrid	ND	ND	Impaired	Reduced, <5%	No
PA14		Yes (43)	Yes (43)	Dispersion (11)	Yes	Yes (11)
dt*dspS_PA14_*	Membrane-bound sensor/response regulator hybrid	ND	ND	Impaired	Reduced	No

^*a*^ Based on brightfield microscopy; %, percent of microcolonies with void formation.

^*b*^ ND, not determined.

^*c*^ WT, wild-type.

### Biofilms by *P. aeruginosa* mutant strains impaired in their dispersion response to glutamate and nitric oxide are impaired in void formation

**Fig 5:**
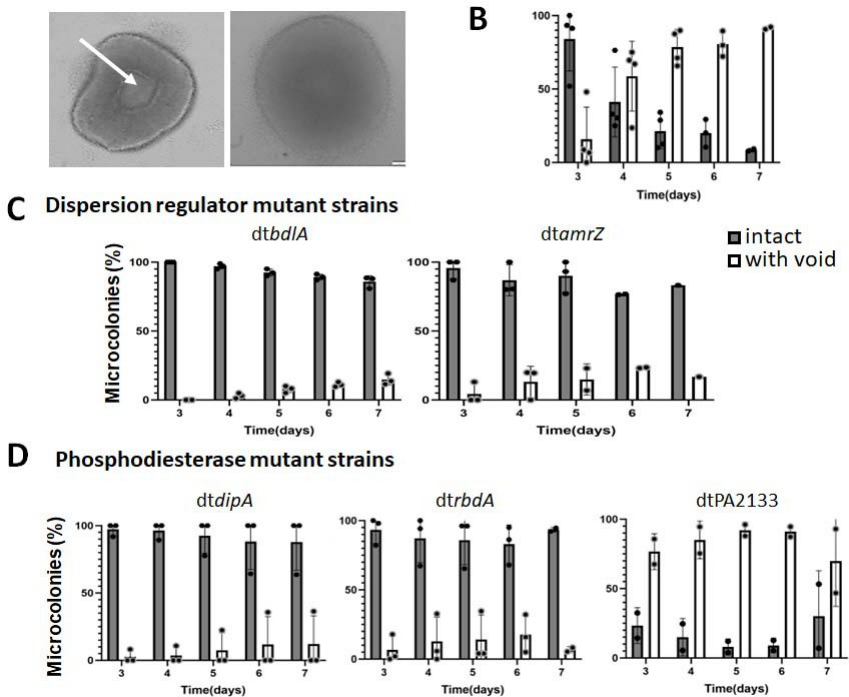
Void formation and native dispersion. Biofilms were grown for up to 7 days in flow cells. (**A**) Representative appearance of microcolonies by *P. aeruginosa* PAO1 post 5 days of growth. Void formation is detectable by brightfield microscopy as central hollowing with bagel-like appearance of microcolonies (see arrow pointing at void in left image); an intact microcolony is shown for comparison in the right image. Size bar, 20 µm. (**B**) Percent of intact microcolonies and those showing void formation at 3, 4, 5, 6, and 7 days of growth of biofilms formed by *P. aeruginosa* PAO1. Percent of intact microcolonies and those showing void formation in biofilms formed by (**C**) dispersion regulator mutant strains dt*bdlA* and dt*amrZ* and (**D**) phosphodiesterase mutant strains dtPA2133, dt*dipA*, and dt*rbdA*, following 3–7 days of growth as biofilms. A total of 50 images of microcolonies were captured daily per strain and evaluated for void formation. Data points represent analysis of image evaluation carried out in triplicate. Error bars represent standard deviations.

Central void formation in biofilm microcolonies is a characteristic consequence of dispersion. A hallmark of native dispersion, voids represent hollowed-out microcolonies due to cells evacuating from the interiors of microcolonies (6, 11, 15, 16). Void formation is detectable by brightfield microscopy as central hollowing and bagel-like appearance of microcolonies. Representative images in Fig. 5A demonstrate the appearance of microcolonies with and without central void formation. Amari et al. (34) demonstrated a link between void formation and *cis*-DA by demonstrating that upon inactivation of *dspI*, the gene encoding the enoyl-CoA synthetase necessary for the production of *cis*-DA, biofilms formed by the Δ*dspI* (PA14_54640, PA0745) mutant were devoid of central voids. Overall, void formation was observed in only 5% of microcolonies of Δ*dspI* mutant biofilms compared with 63% of wild-type PA14 biofilm microcolonies (34). Interestingly, void formation by Δ*dspI* mutant biofilms was restored by complementation with *dspI* and that the Δ*dspI* mutant biofilm retained its ability to disperse in response to *cis*-DA (34). Considering the link between *cis*-DA, native dispersion, and void formation, we reasoned that inactivation of factors contributing to *cis*-DA-dependent dispersion would likewise impair void formation in a manner similar to *dspI* inactivation. As our findings so far suggested that BdlA, AmrZ, and the two PDEs DipA and RbdA (but not PA2133) contribute to dispersion in response to the native dispersion inducer *cis*-DA, the respective mutant strains were selected to determine whether they naturally formed central voids within microcolonies of biofilms grown in flow cells under continuous flow conditions.

To evaluate central void formation, biofilms formed by *P. aeruginosa* PAO1 were monitored by brightfield microscopy over the course of 7 days of growth for void formation. While little void formation was detectable 1–3 days post-inoculation, 46% of wild-type PAO1 biofilm microcolonies demonstrated void formation 4 days post-inoculation, with the percentage increasing to 90% following 7 days of biofilm growth (Fig. 5B). Unlike that of the wild type, void formation was significantly reduced in biofilms formed by *bdlA* and *amrZ* mutant strains, with maximal void formation never exceeding 18% of the microcolonies (Fig. 5C). Likewise, void formation was significantly reduced in biofilms formed by the phosphodiesterase mutants *dipA* and *rbdA*, with average void formation ranging between 3% and 10% over the course of 7 days post-initiation of biofilm formation (Fig. 5D). In contrast, significant void formation was noted for biofilms formed by the phosphodiesterase mutants dtPA2133, with the highest void formation exceeding 94% noted on day 7 (Fig. 5D). Moreover, relative to wild-type biofilms, void formation appeared to be accelerated, as void formation exceeded that noted for wild-type biofilms at days 4 and 5 of biofilm growth (Fig. 5B and D).

Our findings suggested that inactivating *bdlA*, *amrZ*, *dipA*, and *rbdA* abolishes or significantly reduces void formation. Given the link between void formation and native dispersion, our findings indicate that the inactivation of *bdlA*, *amrZ*, *dipA*, and *rbdA* impairs native dispersion, also referred to as biofilm dispersion autoinduction, in continuous cultures of *P. aeruginosa* (Table 1). Taken together, our findings demonstrate that factors required for void formation and, thus, native dispersion (autoinduction) are the same as those required for dispersion in response to exogenously added *cis-*DA or in response to glutamate or nitric oxide (Table 1).

### Previously reported dispersion cue sensory proteins do not contribute to *cis*-DA signal sensing

Dispersion cues such as glutamate or nitric oxide are perceived by membrane-bound sensory proteins. For example, NicD has been reported to perceive nutrient cues (amino acids/sugars) while NbdA senses nitric oxide (Table 1) (23, 24, 43, 59). In contrast, MucR has been reported to sense both nutrients and nitric oxide as dispersion cues (Table 1). No sensory protein to perceive heavy metals or *cis*-DA has yet been identified. Given the apparent redundancy in dispersion cue sensing of the known sensory proteins, we evaluated the biofilms’ response formed by mutants inactivated in genes encoding the dispersion cue sensory proteins NicD, NbdA, and MucR to *cis*-DA. The respective mutant biofilms dispersed in response to *cis*-DA (Fig. 4C; Fig. S3C), with quantitative analysis confirming the mutant strains to disperse in a manner comparable to wild-type biofilms (Fig. 4C). The findings strongly suggested that none of the previously identified sensory proteins contributes to *cis*-DA signal sensing and that *cis*-DA is perceived via sensory protein other than NicD, NbdA, or MucR.

### The two-component sensor PA4982 does not play a role in *cis*-DA-dependent dispersion

**Fig 6:**
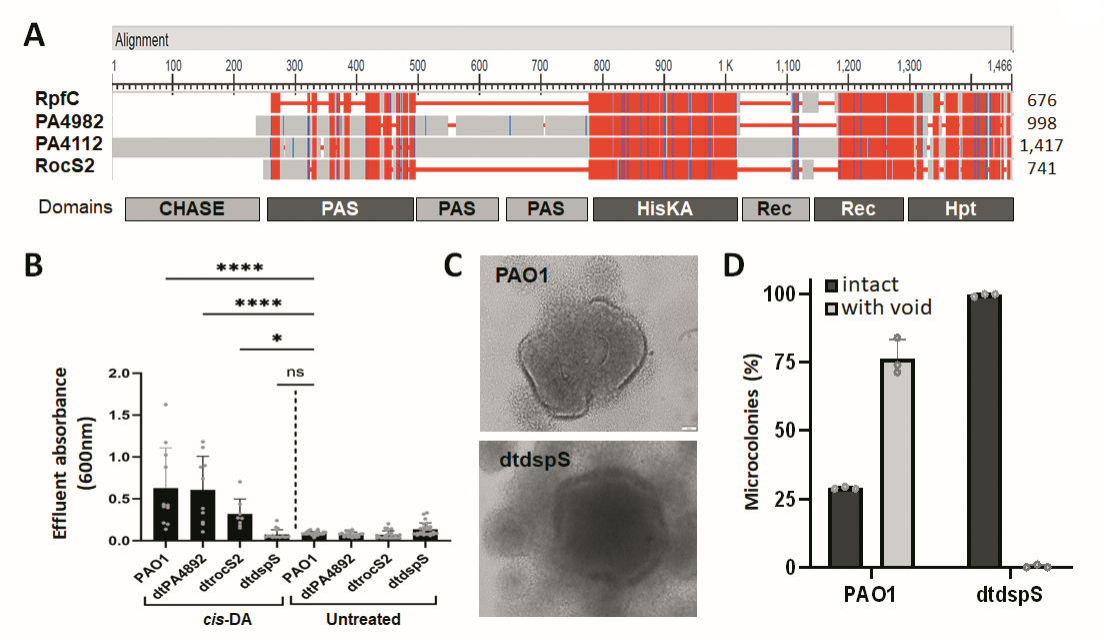
The sensor/response regulator hybrid DspS is required for the cis-DA-induced dispersion response by *P. aeruginosa* PAO1. (**A**) Sequence alignment of RpfC by *Xanthomonas campestris* and the *P. aeruginosa* two-component sensory proteins PA4982, PA4112 (DspS), and PA3044 (RocS2). Red indicates amino acid residues that are similar while purple indicates identical residues. Grey represents amino acid residues that are dissimilar or unique in one sequence. Numbers to the right indicate the total number of residues per indicated protein. Boxes below the alignment indicate the approximate location and identity of domains. Domains shaded in light gray are only present in PA4112 (DspS) while domains shaded in dark gray are present in all protein sequences. CHASE, cyclases/histidine kinases associated sensory extracellular domain; PAS, Per-Arnt-Sim domain; HisKA, histidine kinase domain; Rec, receiver domain; Hpt, histidine phosphotransfer domain. (**B**) Biofilms formed by wild-type PAO1 and isogenic mutants inactivated in the previously predicted *cis*-DA sensory protein PA4982, *rocS2* (PA3044), and PA4112 (*dspS*) were grown for 5 days in fivefold diluted VBMM in tube reactors under flowing conditions. Dispersion was induced after 5 days of growth by the addition of 310 nM cis-DA to the growth medium, and the absorbance values of effluents were obtained 12–18 min post-addition of *cis*-DA and of untreated biofilms quantitatively analyzed. * and ****, significantly different from untreated wild-type biofilms (*P* < 0.05 and *P* < 0.001, respectively), as determined by ANOVA followed by a Dunnett’s post-hoc test. ns, not significant. (**C**) Representative brightfield images of biofilms formed by *P. aeruginosa* PAO1 and dt*dspS* grown for 5 days in flow cells under flowing conditions. Size bar, 20 µm. (**D**) Percentage of intact microcolonies and those showing void formation in biofilms formed by *P. aeruginosa* PAO1 and dt*dspS* post 5 days of growth under flowing conditions. A total of 50 images of microcolonies were captured daily per strain and evaluated for void formation. Individual data points represent results of image evaluation carried out in triplicate. Error bars represent standard deviations.

Rahmani-Badi et al. (35) proposed PA4982-PA4983 encoding a two-component system to be involved in *cis*-DA signal sensing, with the sensor encoded by PA4982 being 37.9% identical to RpfC by *Xanthomonas campestris* (Fig. 6A). However, the authors (35) provided no experimental evidence for cis-DA being sensed via PA4982-PA4983. To determine whether PA4982 indeed contributes to *cis*-DA signal sensing to induce dispersion, we next evaluated the dispersion response of biofilms formed by the transposon mutant strain dtPA4982. Exposure of biofilms formed by dtPA4982 to *cis*-DA exhibited a dispersion response similar to that of wild-type biofilms (Fig. 4B and 6B; Fig. S4A), indicating that PA4982 is not involved in *cis*-DA signal sensing or *cis*-DA-dependent dispersion.

### PA4112 encodes the dispersion sensor DspS required for *cis*-DA signal sensing and dispersion

Considering that PA4982 does not play a role in dispersion in response to *cis*-DA, we next screened the *P. aeruginosa* genome for homologs of the DSF sensory protein, RpfC, to identify potential *cis*-DA sensory proteins. The search revealed several two-component sensors and sensor/response regulator hybrids, with the closest matches including PA4892, LadS, SagS, RocS1, PA3044, and PA4112. The two-component sensors with known functions (LadS, SagS, and RocS1) were excluded from the search, leaving PA3044 (30.9% identity to RpfC) and PA4112 with 34.9% identity to RpfC (Fig. 6A). Next, biofilms formed by the respective transposon mutant strains dt*rocS2* and dtPA4112 were evaluated for their response to *cis*-DA. Insertional inactivation of *rocS2* had little to no effect on the dispersion response relative to wild-type biofilms (Fig. 6B; Fig. S4B). In contrast, insertional inactivation of PA4112 impaired the dispersion response to levels comparable to that of untreated biofilms (Fig. 6B; Fig. S4B). The findings suggested that while PA3044 (RocS2) plays no role in *cis*-DA-induced dispersion, PA4112 does. PA4112 is predicted to encode a sensor/response regulator hybrid. We, therefore, named PA4112 DspS for dispersion sensor.

### Inactivation of *dspS* impairs void formation

To further confirm the role of DspS in *cis*-DA signaling and dispersion, we next asked if the inactivation of *dspS* would likewise impair void formation in a manner similar to the inactivation of *dspI* (34) or *bdlA* or *dipA* (Fig. 5C and D). We chose central void formation as an indicator of native dispersion. To evaluate central void formation, flow cell-grown biofilms by *P. aeruginosa* dt*dspS* were grown for 5 days, after which microcolonies were evaluated for void formation. Wild-type biofilms were used as controls. Relative to wild-type biofilms, dt*dspS* mutant biofilms were characterized by larger microcolonies (Fig. 6C). Moreover, biofilms formed by dt*dspS* demonstrated little to no void formation relative to wild-type biofilms (Fig. 6C and D). In fact, while on average, 56% of wild-type PAO1 biofilm microcolonies demonstrated void formation 5 days post-inoculation, none of the 90 *dspS*::IS biofilm microcolonies that were analyzed showed signs of voids (Fig. 6C and D). Using central void formation as an indicator for native dispersion, our findings strongly support the notion of DspS playing a role in native dispersion as well as *cis*-DA-induced dispersion (Table 1).

### The DspS homolog contributes to *cis*-DA-induced and native dispersion of *P. aeruginosa* PA14 biofilms

To further confirm the role of DspS in *cis*-DA-induced and native dispersion, we asked whether the DspS homolog in *P. aeruginosa* PA14, encoded by PA14_10770 and referred to here as DspS_PA14_, likewise contributes to dispersion.

**Fig 7:**
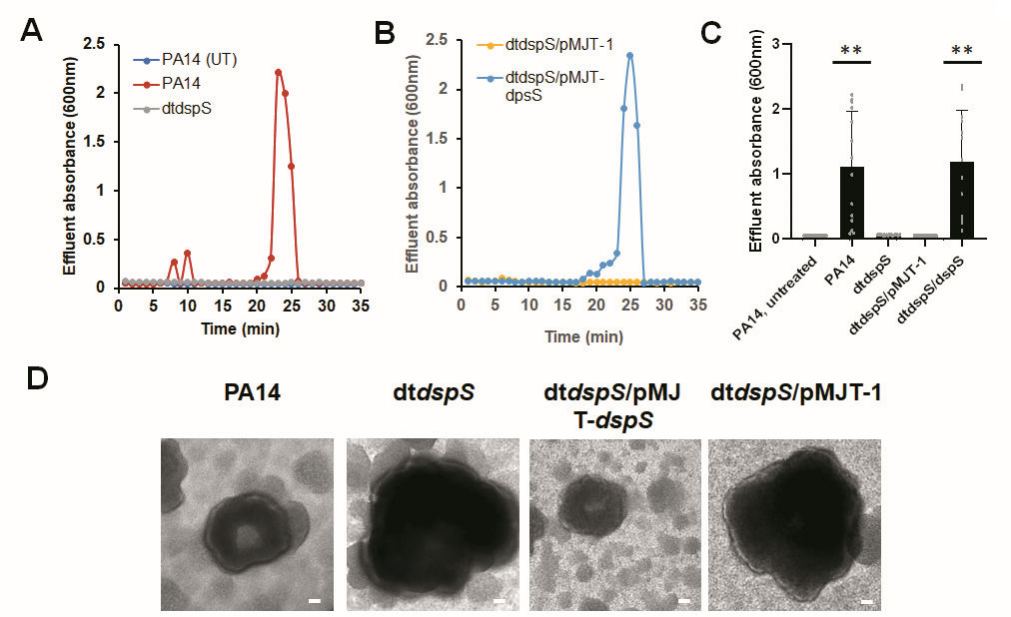
Inactivation of the DspS homolog in *P. aeruginosa* PA14 impairs native dispersion and dispersion in response to cis-DA. Biofilms by *P. aeruginosa* PA14 and dt*dspS*_PA14_ were grown for 5 days in fivefold diluted VBMM in tube reactors under flowing conditions. Dispersion was induced after 5 days of growth by the addition of 310 nM *cis*-DA to the growth medium. (**A**) Dispersion response of PA14 and dt*dspS*_PA14_. UT, absorbance of effluent of untreated wild-type biofilm. (**B**) Dispersion response of biofilms formed by dt*dspS*_PA14_ harboring the empty vector pMJT-1 (dt*dspS*_PA14_/pMJT-1) or the complemented strain dt*dspS*_PA14_/pMJT-*dspS*. (**C**) Quantitative analysis of the dispersion response shown in panels **A and B**. Data represent absorbance values of effluents obtained at 20–25 min post-addition of *cis*-DA. Untreated biofilms were used as controls. Experiments were carried out in triplicate. **, significantly different from untreated wild-type biofilms (*P* < 0.001), as determined by ANOVA followed by a Dunnett’s post-hoc test. (**D**) Representative brightfield images of biofilms formed by *P. aeruginosa* PA14, dt*dspS*_PA14_, and dt*dspS*_PA14_ harboring the empty vector pMJT-1 or pMJT-*dspS*. Biofilms were grown for 5 days in flow cells under flowing conditions. Microcolonies formed by dt*dspS*_PA14_ mutant biofilms remained intact, whereas microcolonies formed by wild type and the complemented mutant showed native biofilm dispersion, as evidenced by the formation of void at the center of the microcolony. Size bar, 20 µm.

We made use of *P. aeruginosa* PA14 and the transposon mutant dt*dspS_PA14_. cis*-DA-induced dispersion was carried out using biofilms of the respective strains grown in tube reactors. While biofilms formed by *P. aeruginosa* PA14 dispersed in response to *cis*-DA, apparent by a sharp increase in the absorbance of the effluent, biofilms formed by dt*dspS_PA14_* failed to do so (Fig. 7A). Instead, the absorbance of the biofilm effluent of this mutant strain was comparable to that of untreated biofilms (Fig. 7A and C). Notably, multicopy expression of *dspS* restored the dispersion phenotype of dt*dspS_PA14_* to wild-type levels (Fig. 7B and C).

We likewise evaluated void formation by *P. aeruginosa* PA14 and dt*dspS_PA14_* biofilms. Biofilms by the respective strains were grown for 5 days, after which microcolonies were evaluated for void formation. The microscopic analysis of microcolonies also indicated wild-type PA14 biofilm microcolonies demonstrating voids. In contrast, void formation was significantly reduced in biofilms formed by dt*dspS_PA14_* (Fig. 7D). Moreover, relative to wild-type PA14 biofilms, dt*dspS_PA14_* mutant biofilms were characterized by overall larger microcolonies (Fig. 7D). The increase in the diameter of dt*dspS_PA14_* microcolonies is consistent with the increase in microcolonies noted for dt*dspS* relative to PAO1 (Fig. 7D). Our microscopic observations of dt*dspS_PA14_* forming more substantial biofilms, with a multicopy expression of *dspS* restoring the biofilm architecture to wild-type levels, were confirmed by COMSTAT analysis (Table 2). Multicopy expression of *dspS* also restored the void formation of dt*dspS_PA14_* biofilms to wild-type levels (Fig. 7D).

**TABLE 2:** Quantitative analysis of biofilm architecture using COMSTAT^*a*^

Strains	Total biomass (µm^3^/µm^2^)	Average biofilm thickness (µm)	Maximum biofilm thickness (µm)
PA14	13.4 (±4.6)	12.3 (±4.7)	36.6 (±6.3)
dt*dspS_PA14_*	29.1 (±6.4)^*b*^	30.3 (±6.8)^*b*^	54.8 (±9.1)^*b*^
dt*dspS _PA14_*/pMJT-*dspS*	14.7 (±3.6)^*c*^	13.9 (±3.6)^*c*^	32.9 (±8.2)^*c*^
dt*dspS _PA14_*/pMJT-1	28.6 (±9.9)^*b*^	28.2 (±10.5)^*b*^	54.0 (±7.3)^*b*^

^*a*^ COMSTAT analysis was carried out from biofilms grown in triplicate (*n* = 3) from at least eight images per replicate.

^*b*^ Significantly different from the wild-type PA14 (*P* < 0.01), as determined by single-variant ANOVA (Dunnett’s post-hoc test).

^*c*^ Significantly different from dt*dspS*_*PA14*_ and dt*dspS _PA14_*/pMJT-1 (*P* < 0.01), as determined by single-variant ANOVA (Dunnett’s post-hoc test).

## DISCUSSION

**Fig 8:**
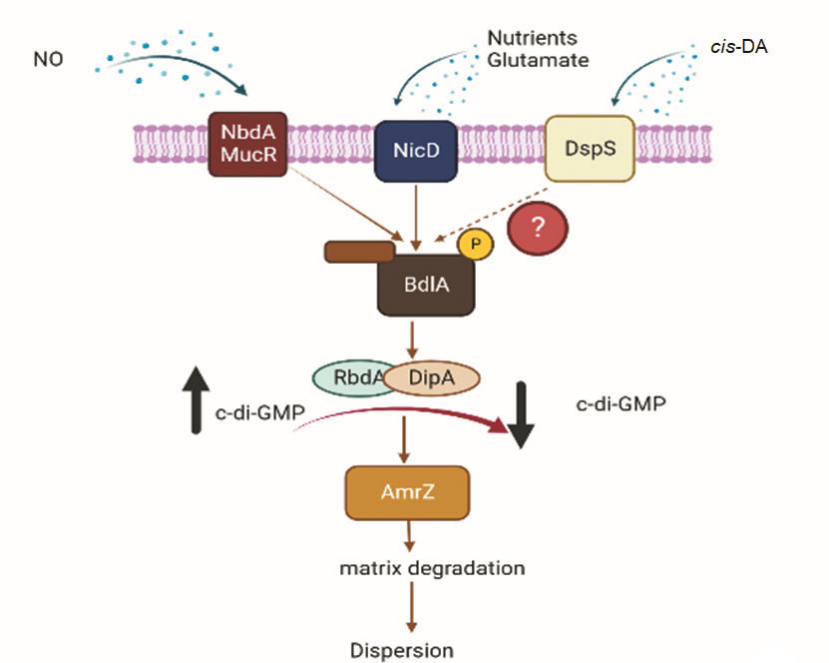
Model of the signaling cascade involved in native dispersion and sensing dispersion cues and signals in *P. aeruginosa.* Dispersion cue receptors NbdA and MucR contribute to nitric oxide sensing (23) while NicD contributes to sensing nutrient cues (glutamate, succinate) (24). This study has identified the sensor/response regulator hybrid DspS as the sensory protein required for *cis*-DA sensing and to be essential for native dispersion and dispersion in response to cis-DA. Sensing of dispersion signals/cues results in non-processive proteolytic cleavage of BdlA, leading to its activation (25). ?, unknown signal relay to BdlA. Once activated, BdlA recruits and activates two phosphodiesterases, DipA and RbdA, which subsequently lower the overall c-di-GMP levels (17, 24, 25). Black arrow pointing up indicates high c-di-GMP levels (but decreased phosphodiesterase activity) while black arrow pointing down indicates increased phosphodiesterase activity but low c-di-GMP levels. AmrZ activates genes encoding matrix-degrading enzymes (30). Matrix degradation contributes to dispersion (53, 54). PA2133 is not included as the findings here do not support a role in native dispersion.

This study aimed to conduct a mechanistic investigation of native and *cis*-DA-dependent biofilm dispersion in *P. aeruginosa*. We approached this question by asking whether native dispersion and dispersion in response to exogenously added *cis*-DA required similar factors/proteins as dispersion in response to the dispersion inducers nitric oxide and glutamate. Our findings suggested that similar to dispersion triggered by nitric oxide and glutamate, dispersion induced by *cis*-DA required the regulatory proteins AmrZ and BdlA as well as two phosphodiesterases, DipA and RbdA (Fig. 4; Table 1), but not the phosphodiesterase PA2133 (Fig. 4; Table 1). Likewise, native dispersion, which occurs without the exogenous addition of a dispersion inducer and is apparent by the formation of central voids (Fig. 5), requires the same regulatory proteins as *cis*-DA-induced dispersion. Our findings also indicated that none of the previously identified sensory proteins contributed to *cis*-DA signal sensing. These included NicD, which has been previously reported to sense nutrient cues (24), NbdA, which perceives nitric oxide (23), and MucR, which has been shown to respond to both nutrient cues and nitric oxide (23). Overall, our findings demonstrated that while the sensing of *cis*-DA and dispersion cues such as nitric oxide and glutamate are distinct, the downstream mechanisms leading to the liberation of biofilm cells and, thus, dispersion rely on a shared pathway. These findings are summarized in Fig. 8 and are supported by our observations that both native and *cis*-DA-induced dispersion coincided with reduced cellular c-di-GMP levels as well as increased expression of genes encoding matrix-degrading enzymes, including *pelA*, *pslG*, *endA*, and *eddA* (Fig. 2). Additional evidence of native and induced dispersion requiring similar factors stems from the observations that dispersion was not enhanced when induced with *cis*-DA plus nitric oxide compared with induction with *cis*-DA alone (Fig. 3). This contrasted with the dispersion response obtained following exposure to pyruvate depletion conditions which was found to be enhanced upon co-incubation with nitric oxide (Fig. 3). The findings have two important implications. First, pyruvate depletion is distinct from *cis*-DA- and nitrous oxide/glutamate-induced dispersion. This finding is supported by pyruvate depletion-induced dispersion being independent of BdlA, RbdA, and DipA (58). Second, the efficacy of the dispersion response can be enhanced by combining pyruvate depletion with nitric oxide addition, while *cis*-DA-induced dispersion could not be enhanced when combined with nitric oxide. In support of our conclusion, Barraud et al. noted that following an initial dispersion response, repeated exposure of biofilms to dispersion-inducing conditions does not result in enhanced dispersion. It is of interest to note that little is known about the mechanism of dispersion induced by starvation or oxygen-limiting conditions. Considering the similarities between dispersion induced by the above two conditions, it is likely that such a dispersion response is also dependent upon proteins described to contribute to *cis*-DA-induced *P. aeruginosa* biofilm dispersion. However, additional research will be required to determine whether dispersion induced by starvation or oxygen-limiting conditions shares the same regulatory mechanism as native dispersion.

In this work, we have furthermore provided evidence that the two-component sensor/response regulator hybrid DspS (PA4112, PA14_10770) is required and essential for native biofilm dispersion in response to the cell-to-cell signaling molecule *cis*-DA (Fig. 6 and 7). DspS from *P. aeruginosa* PAO1 and *P. aeruginosa* PA14 are 99.4% identical (Fig. S5). DspS homologs are prevalent among *Pseudomonas* sp., with pseudomonas.com indicating the presence of 846 group orthologs. However, while DspS demonstrates up to 40% homology to other two-component sensors, including the *X. campestris* RpfC (less than 40% homology), homologs with higher homology (above 60%) appear to be limited to *Pseudomonas* sp. The gene *dspS* is predicted to be encoded by a single gene operon of 4,254 bp, with DspS having a molecular mass of 153.9 kDa (61). DspS is composed of an N-terminal CHASE (cyclases/histidine kinases associated sensory extracellular) domain bracketed by probable transmembrane helices, followed by two PAS (Per-Arnt-Simt) domains, a HisKA (histidine kinase) domain and a C-terminal Rec domain (61) (Fig. 6A). This finding is in contrary to what has been previously proposed by Rahmani-Badi et al. who proposed that PA4982 acts as the sensory protein for signal sensing in the native dispersion response of *P. aeruginosa* (35).

DspS from *P. aeruginosa* is 34.9% identical to the DSF sensor kinase RpfC in *X. campestris* (108), which is not surprising considering the evolutionary relatedness of these two organisms and considering that both proteins detect structurally homologous small fatty acid signaling molecules. However, the two proteins differ in their domain composition, with RpfC harboring an N-terminal PAS sensory input domain composed of five transmembrane-spanning helices with periplasmic and cytoplasmic loops of less than 20 amino acids, a HisKA domain, and a Rec domain, followed by a C-terminal histidine phosphotransfer (HPT) domain (37, 38). The specific residues responsible for DSF binding by RpfC are not known. Considering that CHASE domains have been implicated in sensing diverse types of small molecules (62), it is likely that this periplasmic sensory domain of DspS is involved in *cis*-DA signal sensing. Additional research will be necessary to elucidate the domain and specific residues responsible for *cis*-DA binding by DspS.

In summary, our findings demonstrate that mechanistically, native dispersion induced by *cis*-DA is similar to dispersion induced by nitric oxide or glutamate, with both types of dispersion requiring the action of BdlA, AmrZ, DipA, and RbdA (Fig. 8; Table 1), leading to the modulation of c-di-GMP and biofilm matrix degradation. Native and induced dispersion, however, differ in the membrane-bound sensory proteins required for dispersion cue sensing (Fig. 8; Table 1). Previous findings indicated nitric oxide to be perceived by NbdA and MucR, while NicD senses nutrient cues including glutamate. Here, we report for the first time that *cis*-DA is sensed by DpsS, with DspS being essential for dispersion in response to exogenously perceived *cis*-DA and the native dispersion response by *P. aeruginosa* biofilms.

Considering the promise that dispersion holds as a potential anti-biofilm treatment strategy (63, 64), our findings provide a path for manipulating native dispersion to disrupt existing biofilms either independently or in combination with antimicrobial compounds. Alternatively, a dispersion signal may be used to prevent a dispersion response in order to reduce the potential health risks associated with the unwanted release of bacteria within a host (13).

## MATERIALS AND METHODS

### Bacterial strains, plasmids, media, and growth conditions

Bacterial strains and plasmids used in the present study are listed in Table 3. The PAO1 transposon mutants were obtained from the sequence-verified two-allele library (65). The PA14 transposon mutants were obtained from the sequence-verified PA14 library (66). *Pseudomonas aeruginosa* PAO1 and *Pseudomonas aeruginosa* PA14 were utilized as parental strains. Planktonic cultures were grown in Lennox broth (LB) or Vogel and Bonner citrate minimal medium at 37°C and 220 rpm. Biofilms were grown as indicated below.

**TABLE 3:** Strains and plasmids used in this study

Strain or plasmid	Relevant genotype or description	Source
Strains		
*Pseudomonas aeruginosa* PAO1		
PAO1	Wild type	B. H. Holloway
dt*nicD*	PAO1; PA4929::IS*lacZ*; Tet^R^	(65)
dt*mucR*	*mucR*::Gm	(23)
dt*nbdA*	*nbdA*::Tc	(23)
dt*dipA*	PAO1; PA5017::IS*lacZ*; Tet^R^	(65)
dt*rbdA*	PAO1; PA0861::IS*lacZ*; Tet^R^	(65)
dtPA2133	PAO1; PA2133::IS*lacZ*; Tet^R^	(65)
dt*bdlA*	*ΔbdlA* in PAO1, Km^R^	(42)
dt*amrZ*	WFPA205; PAO1 dtamrZ::tet; *amrZ* replaced with omega tetracycline cassette; Tet^R^	(30)
dtPA4982	PAO1; PA4982::IS*lacZ*; Tet^R^	(65)
dt*rocS2*	PAO1; PA3011::IS*lacZ*; Tet^R^	(65)
dt*dspS*	PAO1; PA4112::IS*lacZ*; Tet^R^	(65)
*Pseudomonas aeruginosa* PA14		
PA14	Wild type	(67)
dt*dspS_PA14_*	PA14 10770::MAR2xT7; Gm^r^	(66)
Plasmids		
pCdrA::*gfp*(ASV)	pUCP22Not-P_cdrA_-RBS-CDS-RNase III-*gfp*(ASV)-T_0_-T_1_, Amp^R^, Gm^R^	(49)
pMJT-1	*araC*-P_BAD_ casette of pJN105 cloned into pUCP18, Amp^r^ Cb^r^	(68)
pMJT-*dspS_*His	His-tagged *dspS* cloned into pMJT-1 using primers; Cb^r^	This study

### Biofilm growth

To extract RNA or evaluate dispersion, biofilms were grown for 5 days under continuous flow conditions in biofilm tube reactors (1-m-long size 14 silicone tubing, Masterflex, Cole Parmer Inc.) with an inner surface area of 25 cm^2^ at a flow rate of 0.2 mL/min, using fivefold diluted VBMM medium (6, 15). To visualize the biofilm architecture and evaluate void formation, biofilms were grown in flow cells (glass surface, BioSurface Technologies) at a flow rate of 0.2 mL/min, using a fivefold diluted VBMM medium. Biofilms were subjected to microscopy analysis 3, 4, 5, 6, and 7 days post-initiation of biofilm growth, as described below.

### Induced (exogenous) biofilm dispersion

To evaluate the dispersion response of biofilms formed by *P. aeruginosa* wild-type and mutant strains in response to exogenously added dispersion cues such as nitric oxide, glutamate, and *cis*-DA, dispersion assays were performed using biofilms grown in tube reactors for 5 days. Dispersion of 5-day-old biofilms was induced by the sudden addition of *cis*-DA (310 nM), glutamate (18 mM), or sodium nitroprusside (500 µM) to the growth medium, as previously described (21, 34, 42). Sodium nitroprusside was used as a source of nitric oxide. Where indicated, dispersion was also induced by a combination of *cis*-DA (310 nM) and sodium nitroprusside (500 µM). As *cis*-DA is dissolved in 0.31% ethanol as carrier solution (11, 34), control biofilms were also challenged with the carrier solution to determine if the solution itself induced a dispersion response. Untreated biofilms were used as negative controls. Dispersed cells were collected from the tube reactor effluents into 96-well microtiter plates at 1-min intervals for up to 35 min. The absorbance of the biofilm effluents was assessed by spectrophotometry at 600 nm. Individual absorbance values of effluents, collected in 1-min intervals, were plotted over time to evaluate the biofilm effluents for spikes, sharp increases in the absorbance of the effluent that are indicative of positive dispersion response. Dispersion events were characterized by an increase in the effluent optical density, with the absorbance being at least two times greater than the baseline. Positive dispersion responses were detectable 15 to 25 min post-exposure to dispersion cues. The absorbance of the effluents collected 15 to 25 min post-exposure to dispersion cues was used for the quantitative analysis of the dispersion response. The absorbance of effluents obtained from untreated biofilms was used as negative control. Dispersion was furthermore evaluated by determining the number of viable cells present in the biofilm effluent, with effluents collected 15 to 25 min post-exposure to dispersion cues. Effluents obtained from untreated biofilms were used as a control. Effluents were washed with saline (0.85% NaCl), homogenized, diluted in saline, and spread plated onto LB agar. Viable cell counts were analyzed following overnight incubation at 37°C. All experiments were done at least in triplicate, with each biological replicate consisting of four technical replicates.

### Native (endogenous) dispersion and void formation

To evaluate native (endogenous) dispersion, biofilms formed by *P. aeruginosa* wild-type and mutant strains under flowing conditions in flow cells were viewed by brightfield microscopy 3, 4, 5, 6, and 7 days post-initiation of biofilm growth using an Olympus BX60 microscope and 20× and 50× UPlanF Olympus objectives. A total of 50 images were captured per time point using a ProgRes CF camera (Jenoptik, Jena, Thuringia, Germany) and processed with ProgRes CapturePro 2.7.7 software. Microcolonies were evaluated for the presence of voids, made apparent by central hollowing and the bagel-like appearance of microcolonies. Experiments were done in triplicate using biological replicates.

### Pyruvate depletion induced dispersion

Dispersion induced by pyruvate-depleting conditions was carried out as described by Goodwine et al. (58). Briefly, *P. aeruginosa* biofilms were grown in a 24-well plate system containing 250 µL fivefold diluted LB medium per well. Wells were inoculated with 10 µL of OD-adjusted (OD at 600 nm of 0.1) overnight cultures. Biofilms were subsequently allowed to grow at 37°C while shaking at 220 rpm, with 24-well plates being kept at a 30° angle. The medium was exchanged every 12 h for 5 days. Following 5 days of biofilm growth, dispersion was induced by the addition of 20 mU pyruvate dehydrogenase (Sigma) in the presence of 2 mM CoA (Sigma), 2 mM β-NAD^+^ (Sigma), 20 µM thiamine pyrophosphate (TPP) (Sigma), and 50 µM magnesium sulfate (MgSO_4_) in fivefold diluted LB. Biofilms were exposed to PDH plus cofactors for a total of 16 h. Additionally, dispersion was induced by exposing biofilms to PDH plus cofactors in the presence of sodium nitroprusside (500 µM) as a source of nitric oxide. Control biofilms were exposed to cofactors alone. Dispersion was evaluated by determining the number of viable cells in the supernatant. Supernatants were washed with saline (0.85% NaCl), homogenized, diluted in saline, and spread plated onto LB agar. Viable cell counts were analyzed following overnight incubation at 37°C. All experiments were performed in at least biological triplicate using two to four technical replicates.

### Quantification of c-di-GMP

Relative c-di-GMP levels of planktonic cells, 5-day-old biofilms grown under flowing conditions in tube reactors, and dispersed cells were determined using a fluorescence-based assay that takes advantage of the c-di-GMP-responsive *cdrA* promoter fused to unstable GFP [P*cdrA*::gfp(ASV)] (49). Biofilms were collected into 0.85% saline, and the resulting suspension was homogenized to ensure disaggregation. Planktonic cells were grown to exponential phase in the absence/presence of nitric oxide and glutamate and washed using saline. SNP was used as a source of nitric oxide. Dispersed cells were obtained as described above, washed with saline, and homogenized. The absorbance (600 nm) and fluorescence (GFP: 485 nm/535 nm; nm) of planktonic, dispersed, and biofilm cells were measured in a 96-well black clear-bottom microtiter plate (Greiner Bio-One) using a SpectraMax i3× plate reader (Molecular Devices). To ensure correlation between absorbance (600 nm) and fluorescence, measurements were also taken of serially twofold diluted samples. Quantifications were performed in triplicate using biological replicates, and the fluorescence unit from GFP was normalized to absorbance.

### RNA extraction and quantitative reverse transcriptase PCR

To obtain RNA from biofilms, wild-type and mutant strains were grown in biofilm tube reactors in a fivefold diluted VBMM medium. Following 5 days of growth, biofilm cells were collected directly into equal volumes of RNA Protect (Qiagen). Isolation of mRNA and cDNA synthesis was carried out as previously described (69–71). qRT-PCR was performed using the BioRad CFX Connect Real-Time PCR Detection System (BioRad) and SsoAdvanced SYBR Green Supermix (BioRad) with oligonucleotides listed in Table 4. *cysD* was used as a control. Relative transcript quantitation was accomplished by first normalizing transcript abundance (based on the threshold cycle value [Ct]) to *cysD* followed by determining transcript abundance ratios. Melting curve analyses were employed to verify specific single-product amplification.

**TABLE 4:** Oligonucleotides used in this study

Oligonucleotide	Sequence
qRT-PCR	
*cysD*_ qRT_F	CTGGACATCTGGCAATACAT
*cysD*_ qRT_R	TCTCTTCGTCAGAGAGATGC
*pelA*_ qRT_F	GGTGCTGGAGGACTTCATC
*pelA*_ qRT_R	GGATGGCTGAAGGTATGGC
*pslG*_ qRT_F	CACGTAAGGGACTCTATCTGG
*pslG*_ qRT_R	AGGAAGTCTTTCCAGACCAC
*eddA*_ qRT_F	CCGACCAGTCGATCTTCTA
*eddA*_ qRT_R	TCCAGACGAAACGGATATT
*endA*_ qRT_F	GCTTTCCCGTTTGTTTGT
*endA*_ qRT_R	TAGAGCTTCCAGCCGATT
Tn mutant screen	
PA3044-rocS2_For	CATCGTCCAGCTCCACAAC
PA3044-rocS2_rev	GCTTATCTAAGGGCCGGAAC
PA4112-dspS_for	GCATCAGGATGAGGAACGAT
PA4112-dspS_rev	GCATGTCGATCATGTGCTTC
Complementation	
dspS_NheI_For^*a*^	GCGCGCGCgctagcGCCGGCGCAGGGACGGAATATC
dspS_NheI_His_Rev^*b*^	GCGCGCGCgagctcTCAATGGTGATGGTGATGATG

^*a*^ Restriction sites are indicated by nucleotides in lowercase.

^*b*^ His tag is indicated by underlined nucleotides.

### Statistical analysis

For pairwise comparison, a two-tailed Student’s *t*-test assuming equal variance or using single-factor analysis of variance was used. In addition, statistical differences between strains and/or conditions were determined using a one-way ANOVA, followed by a Dunnett’s post-hoc test using Prism5 software (GraphPad, La Jolla, CA, USA). Unless otherwise noted, all experiments were performed at least in triplicate using biological replicates.

## SUPPLEMENTAL MATERIAL

Supplemental figures mbio.02570-23-s0001.pdf Fig. S1 to S5.

ASM does not own the copyrights to Supplemental Material that may be linked to, or accessed through, an article. The authors have granted ASM a non-exclusive, world-wide license to publish the Supplemental Material files. Please contact the corresponding author directly for reuse.
