# Supplementary material for: cis-DA-dependent dispersion by Pseudomonas aeruginosa biofilm and identification of cis-DA-sensory protein DspS
Source: mBio. 2023 Nov 28;14(6):e02570-23. doi: 10.1128/mbio.02570-23 (PMC10746223; doi:10.1128/mbio.02570-23)
Supplement: Supplemental figures — Fig. S1 to S5. [file mbio.02570-23-s0001.pdf]

<u>Supplementary figures</u>
for "*cis*-DA dependent dispersion by *Pseudomonas aeruginosa* biofilm, and identification of *cis*-DA-sensory protein DspS"

Supplementary Figure 1 Page 2

Supplementary Figure 2 Page 3

Supplementary Figure 3 Page 4

Supplementary Figure 4 Page 5

Supplementary Figure 5 Pages 6-7

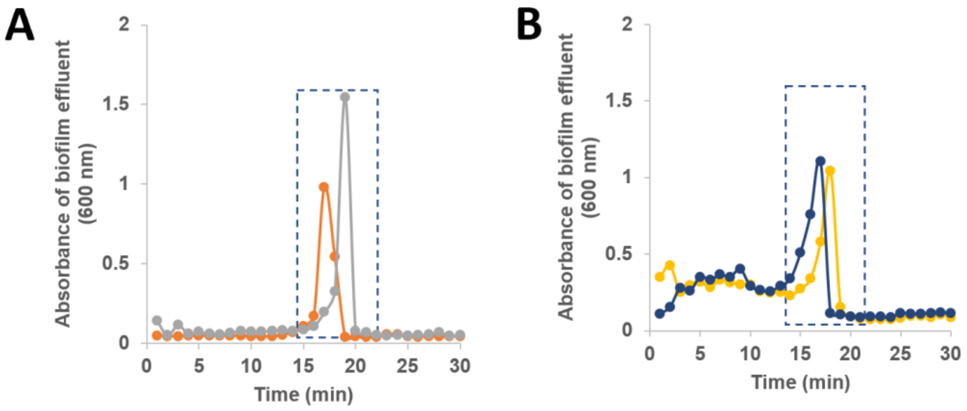

Supplementary Figure 1. Dispersion response of biofilms by *P. aeruginosa* PAO1 following exposure to nitric oxide and glutamate.

Dispersion response of 5-day old *P. aeruginosa* PAO1 biofilms grown in tube reactors under flowing conditions following exposure to (A) nitric oxide (NO) and (B) 18 mM glutamate. Sodium nitroprusside (500 µM) was used as a source of nitric oxide. Post induction of dispersion, effluents from tube reactors were collected for 35 minutes in 1-minute intervals and the absorbance was determined by spectrophotometry at 600nm. Individual data points represent absorbance of effluents collected in 1-minute intervals. Spikes in the absorbance of the effluent are indicative of positive dispersion responses. Dashed box, time frame and corresponding absorbance data used for quantitative analysis of the dispersion response. Dispersion assays were performed in triplicate using four technical replicates. Representative dispersion responses from biological replicates are shown by colored lines.

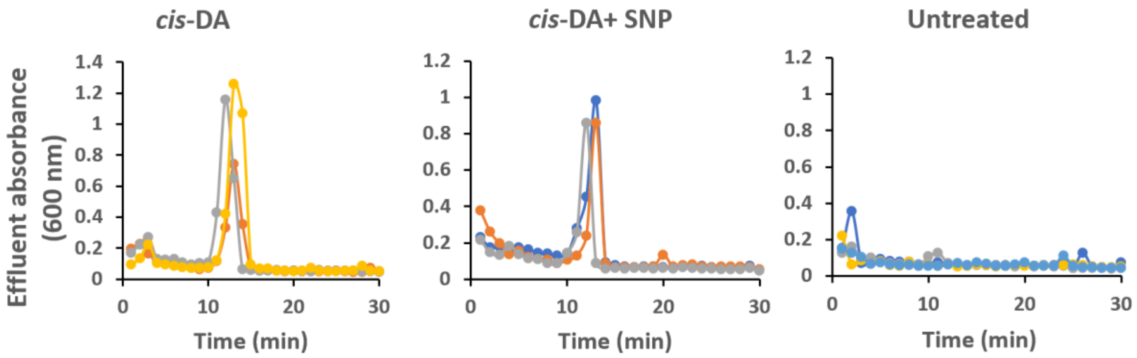

Supplementary Figure 2. Dispersion response of biofilms by *P. aeruginosa* PAO1 following exposure to *cis*-DA or *cis*-DA and nitric oxide.

Dispersion response of 5-day old *P. aeruginosa* PAO1 biofilms grown in tube reactors under flowing conditions following exposure to cis-DA, cis-DA and sodium nitroprusside (SNP), and biofilms left untreated. Sodium nitroprusside (500 µM) was used as a source of nitric oxide. Post induction of dispersion, effluents from tube reactors were collected for 35 minutes in 1-minute intervals and the absorbance was determined by spectrophotometry at 600nm. Individual data points represent absorbance of effluents collected in 1-minute intervals. Spikes in the absorbance of the effluent are indicative of positive dispersion responses. Dispersion assays were performed in triplicate using four technical replicates but only representative dispersion responses from biological replicates are shown by colored lines.

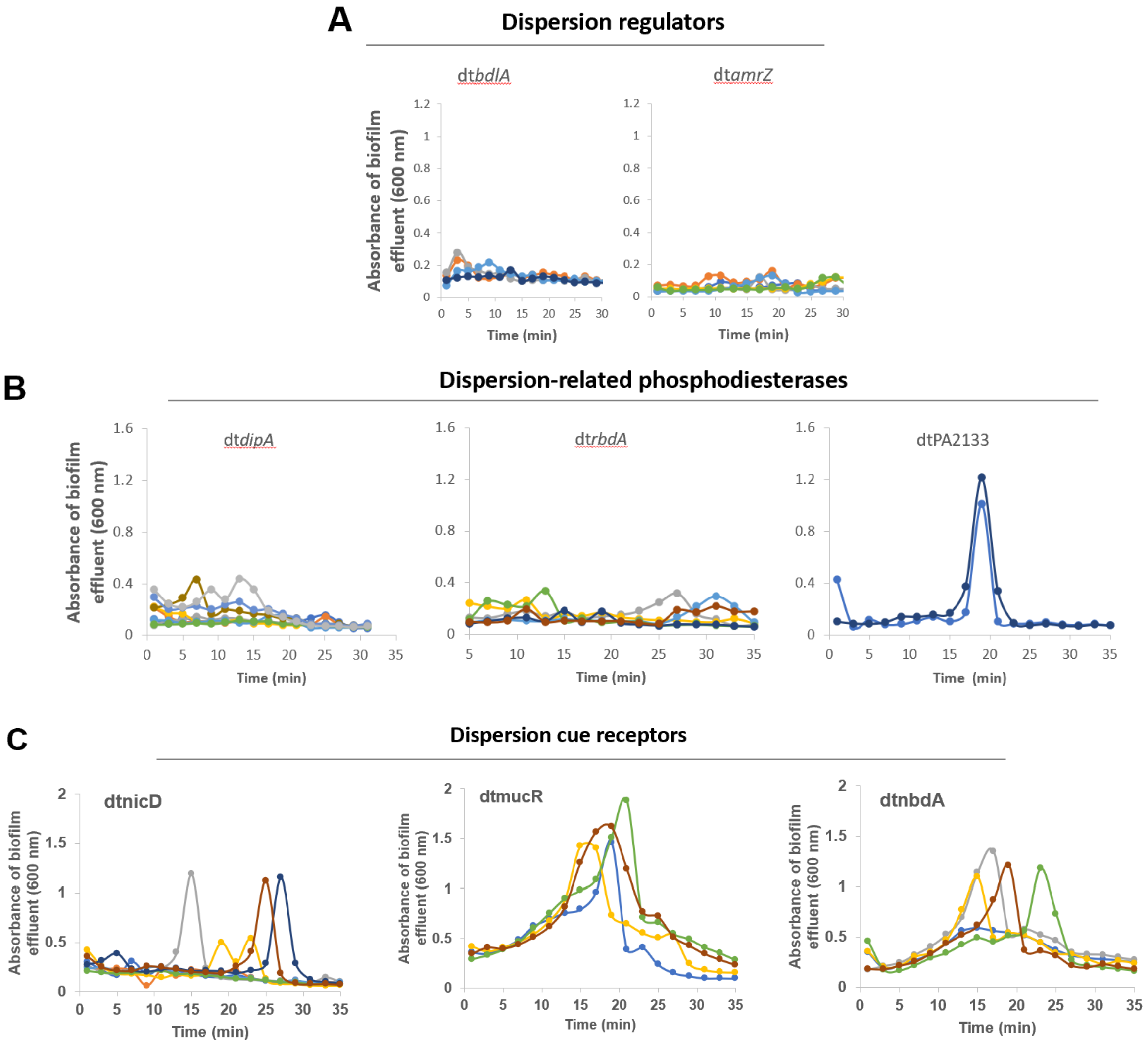

Supplementary Figure 3. Contribution of factors previously reported to contribute to the induced dispersion response, to dispersion in response to the native dispersion signal *cis*-DA

Biofilms were grown for 5 days in 5-fold diluted VBMM in tube reactors under flowing conditions. Dispersion was induced after 5 days of growth by the addition of 310 nM *cis*-DA to the growth medium. Post induction of dispersion, effluent from tube reactors was collected for 30 minutes in 1-minute intervals, and the absorbance was determined by spectrophotometry at 600nm. Individual data points represent absorbance of effluents collected in 1-minute intervals. Spikes in the absorbance of the effluent are indicative of positive dispersion responses. Dispersion response by (A) dispersion regulator mutant strains dt*bdlA* and dt*armZ*, and (B) phosphodiesterase mutant strains dtPA2133, dt*dipA,* and dt*rbdA,* and (C) dispersion cue receptor mutant strains dt*nicD*, dt*mucR,* and dt*nbdA*. Dispersion assays were performed in triplicate using four technical replicates but only representative dispersion responses from biological replicates are shown by colored lines.

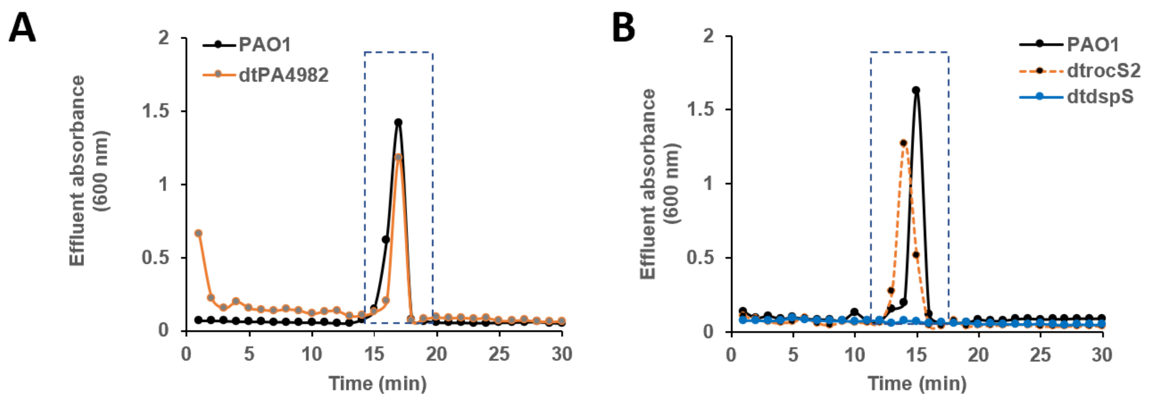

Supplementary Figure 4. Contribution of the *P. aeruginosa* two-component sensory proteins PA4982, PA4112 (DspS), and PA3044 (RocS2) to dispersion in response to exogenous *cis*-DA.

Biofilms were grown for 5 days in 5-fold diluted VBMM in tube reactors under flowing conditions. Dispersion was induced after 5 days of growth by the addition of 310 nM *cis*-DA to the growth medium. Post induction of dispersion, effluent from tube reactors was collected for 30 minutes in 1-minute intervals, and the absorbance was determined by spectrophotometry at 600nm. Individual data points represent absorbance of effluents collected in 1-minute intervals. Spikes in the absorbance of the effluent are indicative of positive dispersion responses. Dispersion response by (A) mutant strain dtPA4982 and (B) mutant strains dt*dspS* (PA4112) and dt*rocS2* (PA3044). Dispersion by PAO1 in response to exogenous *cis*-DA was used as positive control. Dashed box, time frame and corresponding absorbance data used for quantitative analysis of the dispersion response. Dispersion assays were performed in triplicate using four technical replicates but only representative dispersion responses from biological replicates are shown by colored lines.

```
PA4112             1 MNVQQSKFRLSRWAGATLALGLLLSGLGAWGLALVNEQQARMALEREAEL     50
                     ||||||||||||||||||||||||||||||||||||||||||||||||||
PA14_10770         1 MNVQQSKFRLSRWAGATLALGLLLSGLGAWGLALVNEQQARMALEREAEL     50

PA4112            51 LAEAVTRRVELYQYGLRGVRGALLTAGEVHIDRELFRRYSLTRDIDREFP    100
                     |||||.||||||||||||||||||||||.|||||||||||||||||||||
PA14_10770        51 LAEAVIRRVELYQYGLRGVRGALLTAGEAHIDRELFRRYSLTRDIDREFP    100

PA4112           101 GARGFGFIRRVAAADEAGFLRQARADGQPEFRIQQLTPHDGERYVIQYIE    150
                     ||||||||||||||||||||||||||||||||||||||||||||||||||
PA14_10770       101 GARGFGFIRRVAAADEAGFLRQARADGQPEFRIQQLTPHDGERYVIQYIE    150

PA4112           151 PVARNGQALGLDIASEANRREAARAALETGQVRLTGPITLVQASGLRQQS    200
                     ||||||||||||||||||||||||||||||||||||||||||||||||||
PA14_10770       151 PVARNGQALGLDIASEANRREAARAALETGQVRLTGPITLVQASGLRQQS    200

PA4112           201 FLILMPIYRSGITPPPGPQRELEGFGWSYAPLLTGEVLAGLPIDNAAIHL    250
                     ||||||||||||||||||||||||||||||||||||||||||||||||||
PA14_10770       201 FLILMPIYRSGITPPPGPQRELEGFGWSYAPLLTGEVLAGLPIDNAAIHL    250

PA4112           251 ELSDVTGDGAAVPFFVNGAAAPAQRLFGHMLRREIYGRHWQMAFSALPLF    300
                     ||||||||||||||||||||||||||||||||||||||||||||||||||
PA14_10770       251 ELSDVTGDGAAVPFFVNGAAAPAQRLFGHMLRREIYGRHWQMAFSALPLF    300

PA4112           301 VQRLHQPSPRILFLAGSLVSLLLAALVNASALGRLRRRREAATQARLAAI    350
                     ||||||||||||||||||||||||||||||||||.||.||||||||||||
PA14_10770       301 VQRLHQPSPRILFLAGSLVSLLLAALVNASALGRQRRHREAATQARLAAI    350

PA4112           351 VGNSADGIIGVGLDGVISDWNRGAEALFGYREEQAVGRRVVDLLVPPSKE    400
                     ||||||||||||||||||||||||||||||||||||||||||||||||||
PA14_10770       351 VGNSADGIIGVGLDGVISDWNRGAEALFGYREEQAVGRRVVDLLVPPSKE    400

PA4112           401 NEELDILARIARKEQVVSFDTVRRHQDGHLLDVAVTVSPILGPDGGVVGA    450
                     ||||||||||||||||||||||||||||||||||||||||||||||||||
PA14_10770       401 NEELDILARIARKEQVVSFDTVRRHQDGHLLDVAVTVSPILGPDGGVVGA    450

PA4112           451 SKTVRDISAKKAAEARIRELNTGLEHQIAERTAELRRLNVLLGSVLQAAS    500
                     ||||||||||||||||||||||||||||||||||||||||||||||||||
PA14_10770       451 SKTVRDISAKKAAEARIRELNTGLEHQIAERTAELRRLNVLLGSVLQAAS    500

PA4112           501 EVSIITLDRDGIISGFNLGAERMLGYRADEVVGKASPTLLHSERELLARG    550
                     ||||||||||||||||||||||||||||||||||||||||||||||||||
PA14_10770       501 EVSIITLDRDGIISGFNLGAERMLGYRADEVVGKASPTLLHSERELLARG    550

PA4112           551 QELGEEGFRVLVARAEQEGAETREWTYLRKDGSPLSVTLSVTVLRDEAGA    600
                     ||||||||||||||||||||||||||||||||||||||||||||||||||
PA14_10770       551 QELGEEGFRVLVARAEQEGAETREWTYLRKDGSPLSVTLSVTVLRDEAGA    600

PA4112           601 INGYLGIAVDVTEWRNAEREMAAARDQLQMAADVARLGIWRWNLADDSLQ    650
                     ||||||||||||||||||||||||||||||||||||||||||||||||||
PA14_10770       601 INGYLGIAVDVTEWRNAEREMAAARDQLQMAADVARLGIWRWNLADDSLQ    650

PA4112           651 WNERMCEMYGQPLALRDGGLVYEHWRSRLHPEDLERTEASLRAAVEGRGN    700
                     |||||||:||||||||||||||||||||||||||||||||||||||||||
PA14_10770       651 WNERMCELYGQPLALRDGGLVYEHWRSRLHPEDLERTEASLRAAVEGRGN    700

PA4112           701 YDVIFRVVLPDGGIRFIQAGAQVERDADGNPLQVTGINIDITSDQQLQAR    750
                     ||||||||||||||||||||||||||||||||||||||||||||||||||
PA14_10770       701 YDVIFRVVLPDGGIRFIQAGAQVERDADGNPLQVTGINIDITSDQQLQAR    750

PA4112           751 LREAKEQADAASAAKSSFLANMSHEIRTPMNAVLGMLQLARQTDLNERQR    800
                     ||||||||||||||||||||||||||||||||||||||||||||||||||
PA14_10770       751 LREAKEQADAASAAKSSFLANMSHEIRTPMNAVLGMLQLARQTDLNERQR    800
```

```
PA4112          801 DYLDKASSAATSLLGLLNDILDYSKIEAGKLVLEMLPFELEPLMQDLAVV    850
                    ||||||||||||||||||||||||||||||||||||||||||||||||||
PA14_10770      801 DYLDKASSAATSLLGLLNDILDYSKIEAGKLVLEMLPFELEPLMQDLAVV    850

PA4112          851 LSGNQGDKDVEVIFDIDPELPSAVVGDRLRLQQILINLAGNALKFTARGH    900
                    ||||||||||||||||||||||||||||||||||||||||||||||||||
PA14_10770      851 LSGNQGDKDVEVIFDIDPELPSAVVGDRLRLQQILINLAGNALKFTARGH    900

PA4112          901 VLVSLRRLAHDAHLVRLRVLVADTGIGISAEQQQRIFEGFTQAEASTSRR    950
                    ||||||||||||||||||||||||||||||||||||||||||||||||||
PA14_10770      901 VLVSLRRLAHDAHLVRLRVLVADTGIGISAEQQQRIFEGFTQAEASTSRR    950

PA4112          951 FGGTGLGLFICKRLVDLMGGELRVESAPGSGSRFWFDLDLDVAHDQPLRA   1000
                    ||||||||||||||||||||||||||||||||||||||||||||||||||
PA14_10770      951 FGGTGLGLFICKRLVDLMGGELRVESAPGSGSRFWFDLDLDVAHDQPLRA   1000

PA4112         1001 ACPGAGEPLRLLVADDNLVAGELLERTVGALGWRADCVGSGSEAVARVQA   1050
                    ||||||||||||||||||||||||||||||||||||||||||||||||||
PA14_10770     1001 ACPGAGEPLRLLVADDNLVAGELLERTVGALGWRADCVGSGSEAVARVQA   1050

PA4112         1051 AMAEGRRYDVVLMDWRMPDLDGLSAAQLIRQLQGDLPPPMVIMITAYGRE   1100
                    ||||||||||||||||||||||||||||||||||||||||||||||||||
PA14_10770     1051 AMAEGRRYDVVLMDWRMPDLDGLSAAQLIRQLQGDLPPPMVIMITAYGRE   1100

PA4112         1101 VLADARDHSAPPFVDFLTKPVTPKQLADSVLHALHGEQGAPANPPPRPVE   1150
                    ||||||||||||||||||||||||||||||||||||||||||||||||||
PA14_10770     1101 VLADARDHSAPPFVDFLTKPVTPKQLADSVLHALHGEQGAPANPPPRPVE   1150

PA4112         1151 RTQRLRGVRLLVVEDNALNRQVAAELLSSEGARVALADGGLAGVQQVLEA   1200
                    ||||||||||||||||||||||||||||||||||||||||||||||||||
PA14_10770     1151 RTQRLRGVRLLVVEDNALNRQVAAELLSSEGARVALADGGLAGVQQVLEA   1200

PA4112         1201 SVPFDAVLMDMQMPDIDGLEATRRIRADGRFAGLPILAMTANASLADREA   1250
                    ||||||||||||||||||||||||||||||||||||||||||||||||||
PA14_10770     1201 SVPFDAVLMDMQMPDIDGLEATRRIRADGRFAGLPILAMTANASLADREA   1250

PA4112         1251 CLAAGMNDHVAKPIDKERLVLCLLGHLGRSGARGAPATAADAGELVEARG   1300
                    |||||||||||||||||||||||||||||||.||||||.|||||||||||
PA14_10770     1251 CLAAGMNDHVAKPIDKERLVLCLLGHLGRSGDRGAPATTADAGELVEARG   1300

PA4112         1301 DIVGRFGGSLELIVQVLRRFVPDMQDLFAQLERQLGEGDVQGSAATLHTI   1350
                    ||||||||||||||||||||||||||||||||||||||||||||||||||
PA14_10770     1301 DIVGRFGGSLELIVQVLRRFVPDMQDLFAQLERQLGEGDVQGSAATLHTI   1350

PA4112         1351 KGSASTVGASALAGRASELEQALRRADPLRGMEILAGIRLDELRTLCDAC   1400
                    ||||||||||||||||||||||||||||.|||||||||||||||||||||
PA14_10770     1351 KGSASTVGASALAGRASELEQALRRADPRRGMEILAGIRLDELRTLCDAC   1400

PA4112         1401 LLRLQAMFGDVQAESAS   1417
                    |||||||||||||||||
PA14_10770     1401 LLRLQAMFGDVQAESAS   1417
```

Supplementary Figure 5. Alignment of PA4112 (DspS) from *P. aeruginosa* PAO1 and PA14_10770 from *P. aeruginosa* PA14.The protein sequences were pairwise aligned and are 99.4% identical. Vertical lines denote identical amino acid residues while a period indicates dissimilar amino acid residues.
